# Blockchain-Based Traceability System That Ensures Food Safety Measures to Protect Consumer Safety and COVID-19 Free Supply Chains

**DOI:** 10.3390/foods10061289

**Published:** 2021-06-04

**Authors:** Adnan Iftekhar, Xiaohui Cui

**Affiliations:** Key Laboratory of Aerospace Information Security and Trusted Computing, Ministry of Education, School of Cyber Science and Engineering, Wuhan University, Wuhan 430072, China; adnan@whu.edu.cn

**Keywords:** cold supply chain, meat supply chain, food safety, COVID-19, blockchain, hyperledger fabric

## Abstract

The world is facing an unprecedented socio-economic crisis caused by the novel coronavirus infection (COVID-19). The virus is also spreading through the import and export food supply chains. The Chinese authorities have discovered the COVID-19 virus in various imported frozen meat packages. Traceability plays a vital role in food quality and food safety. The Internet of Things (IoT) provides solutions to overseeing environmental conditions, product quality, and product traceability. These solutions are traditionally based on a centralized architecture, which does not guarantee tamper-proof data sharing. The blockchain is an emerging technology that provides tamper-proof data sharing in real-time. This article presents a blockchain-enabled supply chain architecture to ensure the availability of a tamper-proof audit trail. This tamper-proof audit trail helps to make sure that all safety measures are undertaken to minimize the risk of COVID-19 and other bacteria, fungi, and parasites being present in the frozen meat supply chain.

## 1. Introduction

The novel coronavirus was declared a pandemic on 11 March 2020, by the World Health Organization (WHO) [1]. Countries around the world implemented full or partial lockdown to prevent the pandemic from spreading. This resulted in the closure of most businesses, social institutes, and social gatherings around the world. Food supply chains are essential to life. They had to continue normal operations to feed nations worldwide during the pandemic. Researchers have reviewed the COVID-19 pandemic’s impact on the food industry in great detail [2,3,4,5]. This pandemic has disrupted food supply chains worldwide [6]. The center of diseases and prevention in the United States of America reported 16,233 COVID-19 cases and 239 deaths among US workers in 115 meat- and poultry-processing facilities in 19 states [7]. China is one of the biggest importers of frozen meat, mainly from New Zealand, Australia, and Brazil. The Chinese authorities found coronavirus traces on beef and its packaging from Brazil, Bolivia, Australia, and New Zealand [8,9]. Traces of COVID-19 were also found on pork packaging from Argentina [10]. The Chinese authorities sealed the warehouses, canceled the companies’ import licenses, and banned imports from various companies. This has badly impacted labor in China and overseas.

Several rules and regulations have been developed and published in private and government sectors to support the food industry and prevent the infection from spreading. The Chinese State Council also released a circular to prevent COVID-19 infection risk in cold food chains [11]. Traders exporting meat products to China need to double down on ensuring their meat products’ high quality and their record proving COVID-19 prevention compliance [12]. Tracking and traceability is a core component in sustaining the food supply chains. The core goal of supply chain traceability is to track and trace healthy environmental conditions regarding the facilities, workers’ health, and the secure custody of commodities from farm to distributor, to ensure food safety and increase the customer’s trust in their brand.

The vast majority of traditional supply chain information systems are insufficient in providing transparency, and auditability [13]. Several research and development communities and organizations focused on using the Internet of Things (IoT) to provide better information and monitoring facilities to capture the data from the environment and living things to increase food safety standards [14,15]. IoT technology innovations heavily rely on centralized and cloud-based solutions. These solutions are not able to provide enough trusted data and information to cope with the current risks of COVID-19 infection, due to the lack of transparency, data lock-in, and audit-ability [16]. It is very important to maintain reliable, tamper-proof data availability throughout the food supply chain to ensure food safety [17]. It is a big challenge to ensure there are associated data in the food supply chains from origin to destination. These data are essential to prevent COVID-19 and other foodborne illness risks and food integrity issues, and allow for the issuing of various food certificates. Blockchain technology is emerging as the core technology of transparent tamper-proof data sharing.

**Figure 1:**
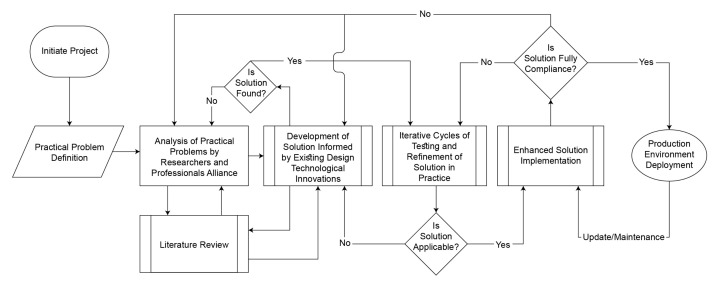
Research Methodology.

We have already published several articles regarding the track-and-trace of the food package from origin to consumer, and deployed blockchain technology in Hubei Government China’s food safety project, with an alliance between food producers and distributors in China and overseas [17,18,19,20]. We designed a research approach with the concept of the mindful use of information technology [17]. The detailed workflow of our developed methodology is given in Figure 1.

## 2. Background Studies

### 2.1. Overview Blockchain Technology

**Figure 2:**
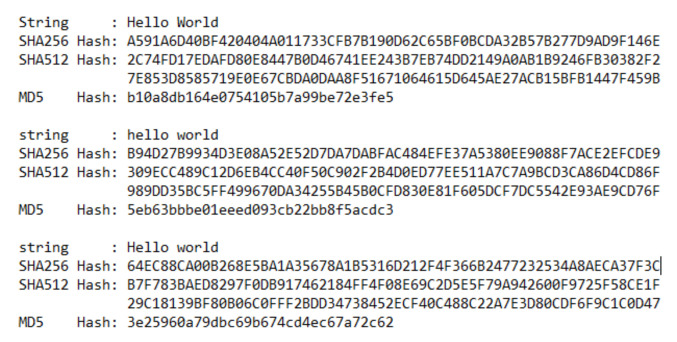
Hash values generated by different Hashing Algorithms.

**Figure 3:**
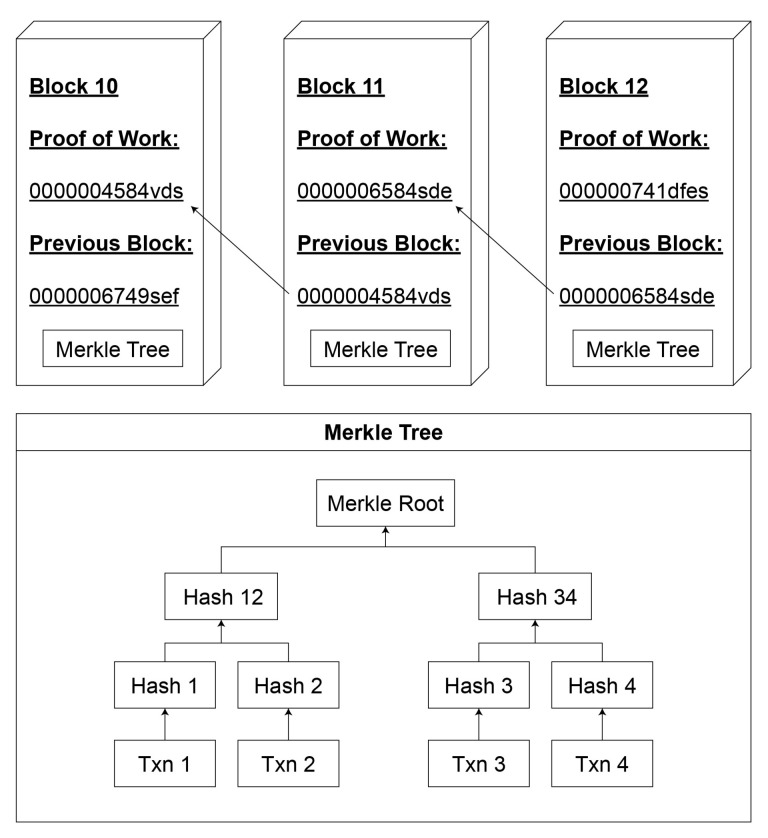
Blocks forming blockchain using hash signature.

Bitcoin originated from the world’s first blockchain-based application [21]. A blockchain is a distributed database consisting of blocks of information integrated with a cryptographic hash function. A hash function processes the given data using a sequence of complex mathematical transformations, resulting in a fixed-length string of characters called a hash value (Figure 2). The hashing process is designed so that even the tiniest change in the data will result in a completely different hash. In Figure 3, we have a chain of three blocks. The use of hash only is not enough to protect the tampering. Current computers calculate billions of hash per second. We can effectively tamper with the block and recalculate all the hashes of the other blocks. This is where proof of work comes in handy. It slows down the creation of new blocks and makes it very hard to tamper with the block, as tampering with one block will also require proof of work for the current and all following blocks [22].

The blockchain’s security comes from its creative use of cryptography, a hashing function, and peer-to-peer networking. When someone joins the blockchain network, it needs to get all the blockchain into the system. The system can verify that everything is in order. When new blocks are created, they are sent over the network to other nodes. Each node then verifies the block validity. If everything is valid, each node adds it to its blockchain. Therefore, all the nodes in the network reach a consensus. They agree upon which block is valid and which block is invalid. Blocks which are tampered with will be rejected by other nodes in the network. If we want to tamper with the public blockchain, we need to calculate all the proof of work, and need more than 50% control of the peer-to-peer network, which is almost impossible to obtain. A detailed study of a blockchain, with its various aspects, architecture and ways of working, can be referred to in [23]. The blockchain-enabled supply chain solutions are shown in Table 1.

### 2.2. COVID-19 and Food Contamination

**Figure 4:**
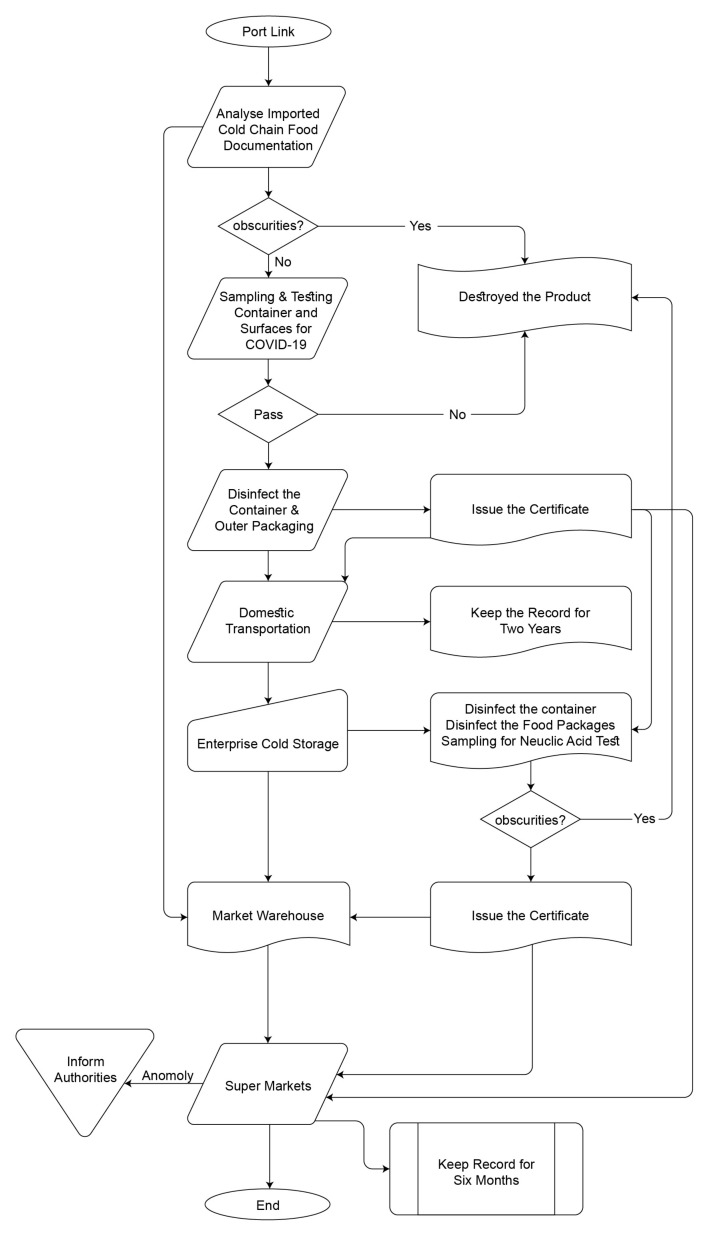
The import process of cold chain products [11].

Zenia et al. have analyzed COVID-19’s impact on the food industry concerning food safety recommendations from various governmental and public organizations [2], and is a basic document from the existing literature in our use case. It is reassuring to state that, despite the worldwide spread of COVID-19, there is no evidence of transmission of this infection via consumption [24,25,26]. Therefore, as stated by public health agencies worldwide, there is no evidence that food poses a risk to public health concerning COVID-19. The main transmission of COVID-19 is through respiratory droplets from the infected [25]. These droplets may quickly fall on the floor and surfaces. The United States Centers for Disease Control and Prevention provided the latest information on COVID-19. The COVID-19 virus survived on cardboard and hard surfaces for up to 72 h in an experimental setting. Thus, it is more likely that the infected food worker will spread the virus from person to person, or through droplets on the surface of the food-processing plant and food packaging [26]. COVID-19 is an issue of occupational safety, and it is necessary to ensure that the workers are healthy and do not contaminate the food through infected droplets on surfaces, food, and packaging [27]. The companies need to implement strict measures for employee health-screening, daily disinfection of surfaces and surroundings, proper use of personal protection equipment by the workers, and effective laundry monitoring. Training and effective communication with the workers are equally important. According to the requirements of the state council’s China “Work Plan for Prevention and Comprehensive Disinfection of Imported Cold Chain Food”, the General Administration of Customs, Ministry of Transportation, Health Commission, and General Administration of Market Supervision are monitoring bodies for the implementation of this plan throughout China citation added [11]. The import process workflow is described with the help of the flow chart in Figure 4.

**Table 1:** Application of Blockchain in the Food Supply Chain.

Authors	Title	Reference
Bumblauskas et al.	A blockchain use case in food distribution: Do you know where your food has been?	[28]
Nir Kshetri	5G in E-Commerce Activities	[29]
Cen et al.	Improving Business Process Interoperability by Shared Ledgers	[30]
Edwards et al.	*Blockchain meets the supply chain*	[31]
Alzahrani et al.	Block-Supply Chain: A New Anti-Counterfeiting Supply Chain Using NFC and Blockchain	[32]
Feng Tian	An agri-food supply chain traceability system for China based on RFID & blockchain technology	[33]
Feng Tian	A Supply Chain Traceability System for Food Safety Based on HACCP, Blockchain & Internet of Things	[34]
Ramundo et al.	State of the art of technology in the food sector value chain towards the IoT	[35]
Daniel et al.	Blockchain application in food supply information security	[36]
Baralla et al.	Ensure Traceability in European Food Supply Chain by Using a Blockchain System	[37]
Chen et al.	Processes, benefits, and challenges for adoption of blockchain technologies in food supply chains: a thematic analysis	[38]
Oslen et al.	Applications, limitations, costs, and benefits related to the use of blockchain technology in the food industry	[39]
Walter G. Johnson	Blockchain Meets Genomics: Governance Considerations for Promoting Food Safety and Public Health	[40]
Mondal et al.	Blockchain Inspired RFID-Based Information Architecture for Food Supply Chain	[41]
Xu et al.	Application of blockchain technology in food safety control: current trends and future prospects	[42]
Feng et al.	Applying blockchain technology to improve agri-food traceability: a review of development methods, benefits and challenges	[43]
Qian et al.	Filling the trust gap of food safety in food trade between the EU and China: An interconnected conceptual traceability framework based on blockchain	[44]
Hao et al.	A Novel Visual Analysis Method of Food Safety Risk Traceability Based on Blockchain	[45]
Adnan et al.	Application of Blockchain and Internet of Things to Ensure Tamper-Proof Data Availability for Food Safety	[17]
Behnke et al.	Boundary conditions for traceability in food supply chains using blockchain technology	[46]
Reuters	Chickens and eggs: Retailer Carrefour adopts blockchain to track fresh produce	[47]
Parshar et al.	Blockchain-Based Traceability and Visibility for Agricultural Products: A Decentralized Way of Ensuring Food Safety in India	[48]

## 3. Blockchain-Based Solution for Importers/Exporters

**Figure 5:**
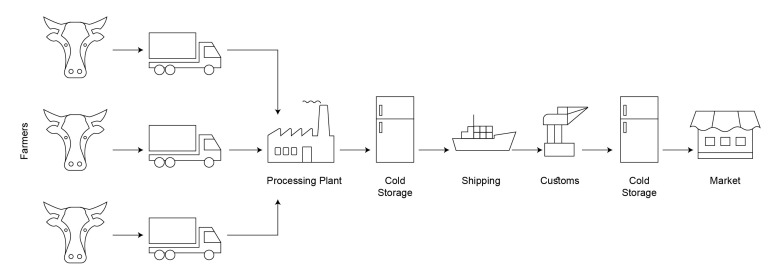
The overview of the beef supply chain.

**Figure 6:**
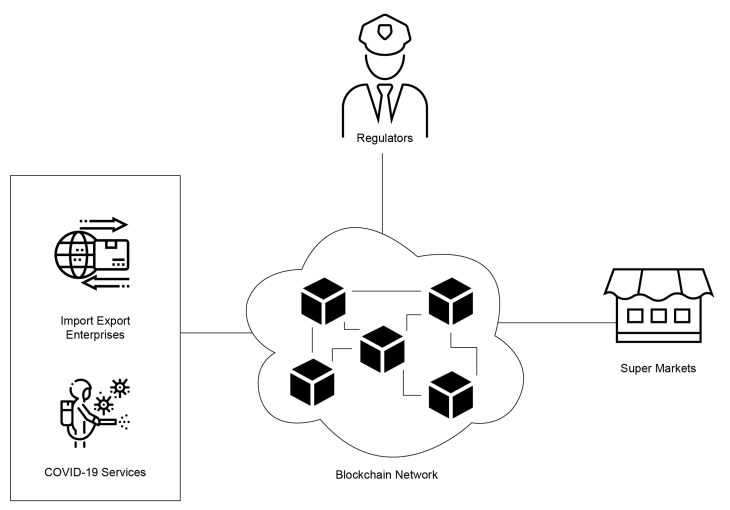
The logical overview of the system.

The beef supply chain is comparatively linear (Figure 5). The supply chain, after the COVID-19 pandemic, included many more actors than before. There are very high chances of lack of trust, due to legal regulations and pandemic control legislation. The blockchain is a single source of truth in a trustless environment. A logical illustration of the blockchain system based on Hyperledger Fabric is given in Figure 6.

**Figure 7:**
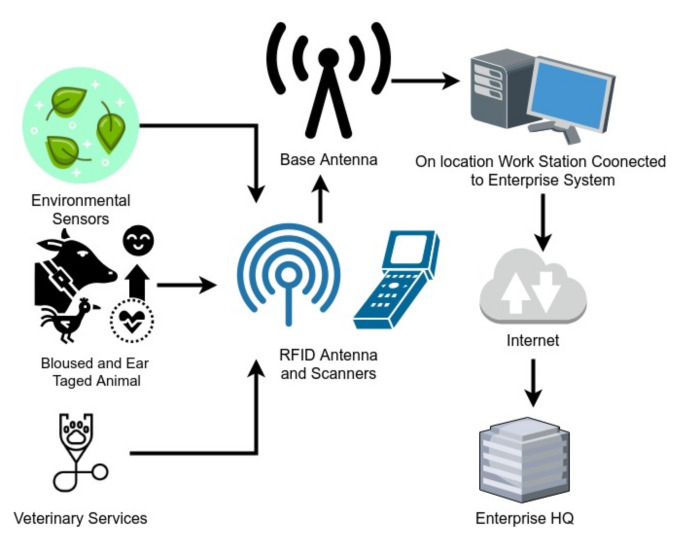
Farm Management System.

The system architecture of our farm management system is shown in Figure 7. Digital data about the feeding and animal breed age and health are digitally available to feed to the blockchain. The processing plant monitors and records every employee’s health, with identification twice a day at the start and end of the shift, and uploads it to the blockchain with a specially designed smart contract. The thermal cameras at the entry–exit and different locations of the plant also continuously monitor the employees’ health. The plants are strictly applying the food safety recommendations. The plant’s livefeed is available to the importers, so they can oversee the plant’s safety conditions. The food packages are carefully packed and completely disinfected with the recommended chemicals, including the container. The enterprise is also responsible for another disinfection at the port, before closing the container and updating the certificate from port authorities on the system. The whole production batch will be returned or destroyed if an anomaly is found in the health of the employees who worked on that specific batch. The flow chart in Figure 4 explainins the domestic operations. All the data will be uploaded on the food safety blockchain system designed for the Hubei Food Safety Authorities.

### 3.1. Blockchain Network Architecture

**Figure 8:**
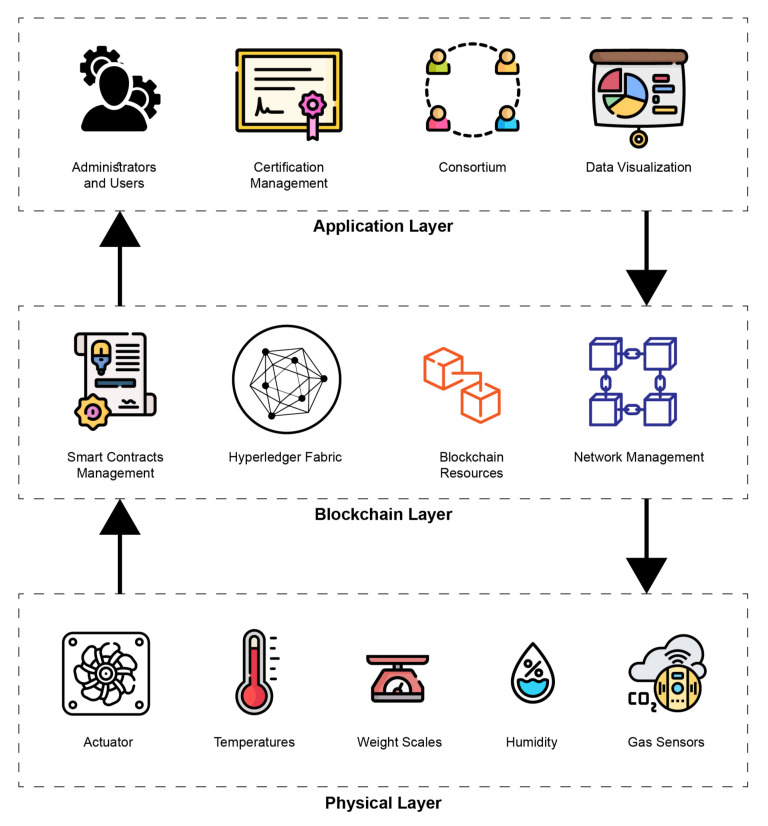
An overview of the Protocol Stack.

Cold chain management requires a large number of reconciliations between different departments. Our proposed use case provides a real-time and tamper-proof trail of transactions to all the essential departments with high integrity. Moreover, it is incredibly flexible and cost-efficient, as it does not require any particular infrastructure or costly servers. Figure 8 offers an overview of our proposed blockchain protocol stack. The Hyperledger Fabric blockchain uses Smart Contracts called Chain Code. The chain code can ensure the transparency of the rules for everyone, and the relevant criteria should trigger interventions. In a blockchain network, the chain codes are the functions that use input in the form of transactions and send notifications to the network’s participants to monitor and notice any violation of the rules. The Hyperledger Fabric is a fully permission-ed network. Each component of the system is required to carry its own identity, including users and operators. Therefore, no violation attempt can be hidden in any way.

The following subsections briefly describe all the layers in the proposed architecture.

### 3.2. Application Layer

The application layer consists of Administrators and Users, Certification Management, Consortium Formation, and Data Visualization. Hyperledger Fabric is a consortium-oriented blockchain platform. The consortium consists of relevant departments and enterprises. According to the government policy, the consortium creates policies to conduct its operations and update its smart contracts, to carry out all the operations.

In the consortium, all the participating entities have an assigned role, with a mutual understanding of the consortium members. The role cannot be changed without the mutual consensus of the participants finthe consortium. The first step is to define the network configurations in the form of policies. With these policies, we can create the first component of the network, which is called the orderer. The orderer serves as the administration point for the rest of the network. The orderer uses the system channel to communicate with the rest of the system components. The orderer needs to configure, with the help of the network configuration. We can retrieve certification from the certification authority to give the identifies to the system components. The certification authority issues an x509 certificate for identity. Each endpoint needs its own identity to recognize the other components from a particular organization or have a specific role in the network. This is why there is usually more than one certification authority in a network. The fabric provides its own CA, which calls the fabric CA. The network configurations depend greatly on generated certificates. This is where the policies define which certificate is needed to perform a particular task.

### 3.3. Blockchain Layer

The second step is to create the channel. For the proposed network, we are going to implement the one channel that is shared by all three organizations. The channel provides a communication mechanism for the members of the consortium. The channel configuration creates a channel, and this configuration is completely separate from that of the network configurations. The organizations will now join the network on the channel as a peer. The peer is a bridge between the fabric network and the real world. Organizations are connected with their peers to gain access to the network. It is also a place where the ledger is stored. This is distributed to all the peers connected to a particular channel. Every channel has one ledger, and every participant has a copy of the ledger. The ledger is logically stored on the channel, but it is stored on the connected peer nodes. At this moment, we have created our channel, and connected all of our participants to the channel. We also have a ledger to store the information. We still need smart contracts to interact with the ledger.

The Hyperledger Fabric blockchain uses Smart Contracts called Chain Code. The chain code can ensure the rules are transparent to everyone, and relevant criteria should trigger food reserve interventions. In a blockchain network, the chain codes are the functions that receive input in the form of transactions and send notifications to the network’s participants to monitor and notice any violation of the rules. The Hyperledger Fabric is a fully permissioned network. Each component of the system is required to carry its own identity, including users and operators. Therefore, no violation attempt can be hidden in any way. The chaincode is deployed on all the peer nodes. The chaincode is not connected to the channel, but it is hosted on the peer nodes. Our network is then ready, and all three organizations have an equal right within the network.

### 3.4. Physical Layer

**Figure 9:**
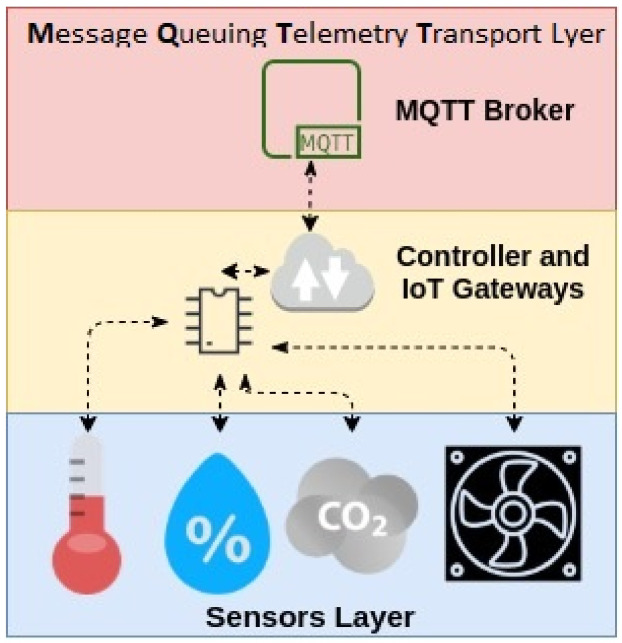
IoT Layer Architecture.

Data have become the most valuable resource, surpassing oil. The internet of things is the pillar of industry 4.0 and agriculture 4.0. The increasing use of different intelligent technologies, such as machine learning, cyber-physical systems and the internet of things, is transforming human life, which is now completely dependent on the data produced by nearby things and information generation. The world’s technological advancements are now directly proportional to global automation, driven by the internet of things and some other physical systems. The systems will provide the foundation of our critical infrastructure, form the basis of emerging and future smart services, and improve our quality of life in many areas. While the internet of things takes care of the connections between objects and machines to the internet, cyber–physical systems are machines in which a mechanism is controlled or monitored by computer-based algorithms. These connected things will interact with each other and make decisions autonomously. One of the data sources that feeds the algorithms comes from the smart sensor systems. The internet of things generally refers to the network of all these sensors, with the ability to collect the data from its environment and feed those data to the algorithms on the processing nodes, globally, through the internet or its local area network (Figure 9).

## 4. Blockchain Network Integration

**Figure 10:**
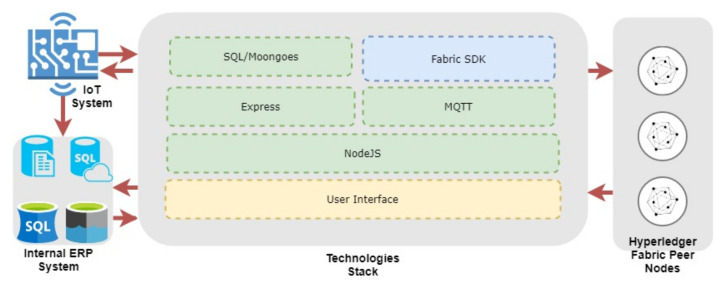
Blockchain Technological Stack to Developed Blockchain Network.

**Figure 11:**
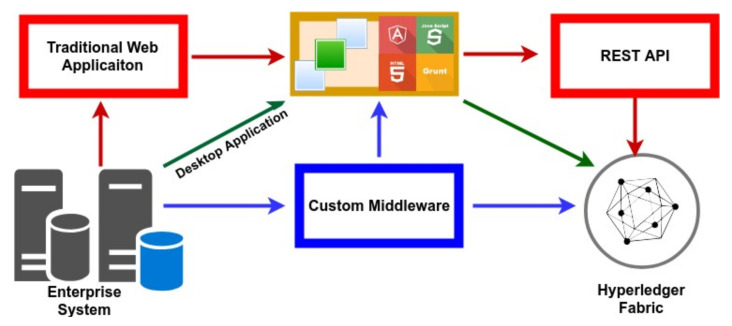
Blockchain Technological Stack to Developed Blockchain Network.

We developed a technological stack to integrate the existing enterprise architecture with a behind-the-scenes blockhain network. Figure 10 refers to our technology stack to develop blockchain-based applications. The technology stack consists of Structured Query Language (SQL), Moongoes, Fabric Software Development Kit (Fabric SDK), Express and Message Queuing Telemetry Transport Broker (MQTT Broker), on top of a NodeJS platform. It provides the functionality required to integrate the blockchain network with the existing enterprise network to load data to/from the blockchain network. The Hyperledger Fabric provides a flexible architecture and Representational State Transfer Application Programmin Interface (REST API) to develop the user interface to interact with the blockchain (Figure 11). The front end of the application can be developed as a traditional web application. A desktop application or a custom middle care can be created using the application development kits provided by Hyperledger Fabric.

## 5. Conclusions

This paper defines a blockchain technology use case and a quick reference guide to design a blockchain network for the food industry. It improves transparency throughout the supply chain and helps reconcile the documentation and required data with legislation authorities to import cold chain products to certify the quality of the final product. The application of a tamper-proof tracing strategy provides reliable traceability and promotes the authenticity of the operations in the supply chain. It establishes a new industry trust mechanism, improves corporate responsibility, and eliminates the tampered practices in the whole supply chain, which also ensures that they eliminate malpractices that risk consumer safety and health. Future studies will focus on developing a common product code and protocols to create a national product recognition database, and develop a system where different blockchains can transfer the data or merge.

## Data Availability

Data sharing is not applicable for this article.
